# Verification and Optimal Control of Context-Sensitive Probabilistic Boolean Networks Using Model Checking and Polynomial Optimization

**DOI:** 10.1155/2014/968341

**Published:** 2014-01-23

**Authors:** Koichi Kobayashi, Kunihiko Hiraishi

**Affiliations:** School of Information Science, Japan Advanced Institute of Science and Technology, Ishikawa 923-1292, Japan

## Abstract

One of the significant topics in systems biology is to develop control theory of gene regulatory networks (GRNs). In typical control of GRNs, expression of some genes is inhibited (activated) by manipulating external stimuli and expression of other genes. It is expected to apply control theory of GRNs to gene therapy technologies in the future. In this paper, a control method using a Boolean network (BN) is studied. A BN is widely used as a model of GRNs, and gene expression is expressed by a binary value (ON or OFF). In particular, a context-sensitive probabilistic Boolean network (CS-PBN), which is one of the extended models of BNs, is used. For CS-PBNs, the verification problem and the optimal control problem are considered. For the verification problem, a solution method using the probabilistic model checker PRISM is proposed. For the optimal control problem, a solution method using polynomial optimization is proposed. Finally, a numerical example on the WNT5A network, which is related to melanoma, is presented. The proposed methods provide us useful tools in control theory of GRNs.

## 1. Introduction

Control of gene regulatory networks (GRNs) is one of the significant topics in the field of systems biology, and is also one of the basics of therapeutic interventions (see, e.g., [1]) in the future. Furthermore, in recent years, the experimental result on control of GRNs has been obtained in [2]. That is, feedback control of synthetic biological circuits has been implemented, and the experimental result in which cellular behavior is regulated by control has been obtained. This result suggests that control methods of GRNs can be realized. Motivated by the above backgrounds, we study a control method of GRNs.

GRNs are in general modeled by ordinary/partial differential equations with high nonlinearity and high dimensionality. In order to deal with such a system, it is important to consider a simple model, and various models such as Bayesian networks, Boolean networks (BNs) [3], hybrid systems (piecewise affine models), and Petri nets have been developed so far (see, e.g., [4] for further details). In control problems, BNs and hybrid systems are frequently used [5–10]. In the hybrid systems-based approach, the classes of GRNs are limited to low-dimensional systems, because the computation time to solve the control problem is too long. In a BN, gene expression is expressed by a binary value (ON or OFF), and dynamics such as interactions between genes are expressed by Boolean functions [3]. In, for example, [11], it is pointed out that a BN is too simple as a model of GRNs. However, there is an advantage that a BN can be relatively applied to large-scale systems. Furthermore, since the behavior of GRNs is stochastic by the effects of noise, it is appropriate that a Boolean function is randomly decided at each time among the candidates of Boolean functions. Thus, a probabilistic Boolean network (PBN) has been proposed in [12], and further, a context-sensitive PBN (CS-PBN) has been proposed as a general form of PBNs [13, 14]. In a CS-PBN, the deciding time is also randomly selected.

In this paper, a CS-PBN is adopted as a model of GRNs, and for CS-PBNs, the verification problem and the optimal control problem are considered. In the verification of PBNs, a solution method using the probabilistic model checker PRISM [15] has been proposed in [16]. However, this PRISM-based method for PBNs has not been extended to that for CS-PBNs. In optimal control of PBNs and CS-PBNs, many results have been obtained so far (see, e.g., [13, 14, 17–24]). In many existing results, state transition diagrams with 2^*n*^ nodes (i.e., 2^*n*^ × 2^*n*^ transition probability matrices) must be computed for a (CS-)PBN with *n* genes. In order to compute state transition diagrams, several issues such as memory consumption must be considered in implementation, and it is desirable to directly use a given Boolean function. The authors have proposed in [22, 23] control methods in which state transition diagrams are not computed. Comparing the methods in [22, 23] with other existing results [13, 14, 17–21, 24], the methods in [22, 23] can relatively handle more large-scale GRNs. The method in [22] can be applied to PBNs and CS-PBNs, but the expected value of a given nonnegative function cannot be evaluated as a cost function (objective function). In the method in [23], the expected value of a given nonnegative function can be used as a cost function, and the optimal control problem is reduced to a polynomial optimization problem. However, this method has been proposed for PBNs, and an extension to CS-PBNs has not been discussed so far. Thus, for verification of CS-PBNs, the PRISM-based method for PBNs [16] is extended to that for CS-PBNs. For optimal control of CS-PBNs, a solution method using polynomial optimization [23] is extended to that for CS-PBNs. Furthermore, the effectiveness of the proposed methods is presented by a numerical example on the WNT5A network, which is related to melanoma. The proposed methods provide us useful tools in control theory of GRNs.

This paper is organized as follows. In Section 2.1, a CS-PBN is explained. In Section 2.2, a solution method for the verification problem is proposed. In Section 2.3, a solution method for the optimal problem is proposed. In Section 3, a numerical example is presented. In Section 4, we conclude this paper.

*Notation.* Let *ℛ* denote the set of real numbers. Let {0,1}^*n*^ denote the set of *n*-dimensional vectors, which consists of elements 0 and 1. For a matrix *M*, *M* ^*⊤*^ denotes the transpose of *M*.

## 2. Materials and Methods

### 2.1. Context-Sensitive Probabilistic Boolean Networks

First, we introduce a probabilistic Boolean network (PBN). Consider the following PBN:

(1) x1(k+1)=f(1)(k,x(k),u(k)),x2(k+1)=f(2)(k,x(k),u(k)),⋮xn(k+1)=f(n)(k,x(k),u(k)),

where $x={\left[\begin{array}{cccc} x_{1} & x_{2} & \cdots & x_{n} \end{array}\right]}^{⊤}\in {\left\{0,1\right\}}^{n}$ is the state (e.g., the expression of genes), $u={\left[\begin{array}{cccc} u_{1} & u_{2} & \cdots & u_{m} \end{array}\right]}^{⊤}\in {\left\{0,1\right\}}^{m}$ is the control input (e.g., the expression of genes), that is, the value of *u* can be arbitrarily given, and *k* = 0,1, 2,… is the discrete time. For a fixed *k* ∈ {0,1,…}, *f* ^(*i*)^ : {0,1,…}×{0,1}^*n*^ × {0,1}^*m*^ → {0,1}^1^ is a given Boolean function consisting of logical operators such as AND (∧), OR (∨), and NOT (¬). In deterministic Boolean networks, *x*(*k* + 1) is uniquely determined for given *k*, *x*(*k*), and *u*(*k*). In PBNs, the candidates of *f* ^(*i*)^(*k*, *x*(*k*), *u*(*k*)) are given, and for each *x*_*i*_, selecting one Boolean function is probabilistically independent at each time. Let *f*_*j*_^(*i*)^(*x*(*k*), *u*(*k*)), *j* = 1,2,…, *l*(*i*), denote the candidates of *f* ^(*i*)^(*k*, *x*(*k*), *u*(*k*)). The probability that *f*_*j*_^(*i*)^(*x*(*k*), *u*(*k*)) is selected is defined by

(2) $$\begin{array}{c} c_{j}^{\left(i\right)}:=\text{Prob}\left(f^{\left(i\right)}\left(k,x\left(k\right),u\left(k\right)\right)=f_{j}^{\left(i\right)}\left(x\left(k\right),u\left(k\right)\right)\right). \end{array}$$

Then, the following relation:

(3) $$\begin{array}{c} \sum_{j=1}^{l(i)}c_{j}^{\left(i\right)}=1 \end{array}$$

must be satisfied. Probabilistic distributions are derived from experimental results, but details are one of the future works. Then, a method for inferring a probabilistic Boolean network will be useful (see, e.g., [25]).

**Example 1**

**Figure 1:**
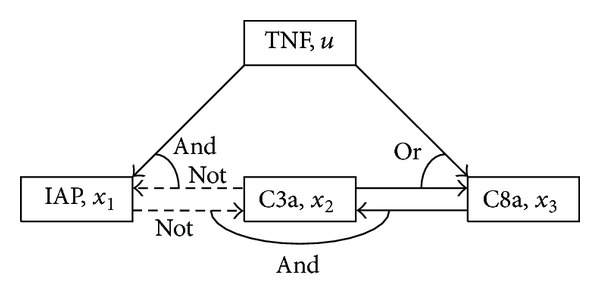
Simplified model of an apoptosis network. Activation (solid), inhibition (broken).

As a simple example, consider the following deterministic Boolean network of an apoptosis network [26, 27] (see also Figure 1):

(4) $$\begin{array}{c} x_{1}\left(k+1\right)=\neg x_{2}\left(k\right)\wedge u\left(k\right),\\ x_{2}\left(k+1\right)=\neg x_{1}\left(k\right)\wedge x_{3}\left(k\right),\\ x_{3}\left(k+1\right)=x_{2}\left(k\right)\vee u\left(k\right), \end{array}$$

where the concentration level (high or low) of the inhibitor of apoptosis proteins (IAP) is denoted by *x*_1_, the concentration level of the active caspase 3 (C3a) by *x*_2_, and the concentration level of the active caspase 8 (C8a) by *x*_3_. The concentration level of the tumor necrosis factor (TNF, a stimulus) is denoted by *u* and is regarded as the control input. Although Boolean dynamics in the above system are synchronous, both synchronous and asynchronous dynamics will be included. From this viewpoint, we consider the following PBN induced by the above system:

(5) $$\begin{array}{c} f^{\left(1\right)}=\{\begin{array}{cc} f_{1}^{\left(1\right)}=\neg x_{2}\left(k\right)\wedge u\left(k\right), & c_{1}^{\left(1\right)}=0.6,\\ f_{2}^{\left(1\right)}=x_{1}\left(k\right), & c_{2}^{\left(1\right)}=0.4, \end{array} \end{array}$$

(6) $$\begin{array}{c} f^{\left(2\right)}=\{\begin{array}{cc} f_{1}^{\left(1\right)}=\neg x_{1}\left(k\right)\wedge x_{3}\left(k\right), & c_{1}^{\left(2\right)}=0.7,\\ f_{2}^{\left(1\right)}=x_{2}\left(k\right), & c_{2}^{\left(2\right)}=0.3, \end{array} \end{array}$$

(7) $$\begin{array}{c} f^{\left(3\right)}=\{\begin{array}{cc} f_{1}^{\left(3\right)}=x_{2}\left(k\right)\vee u\left(k\right), & c_{1}^{\left(3\right)}=0.8,\\ f_{2}^{\left(3\right)}=x_{3}\left(k\right), & c_{2}^{\left(3\right)}=0.2, \end{array} \end{array}$$

where *l*(1) = *l*(2) = *l*(3) = 2, and we give *c*_*j*_^(*i*)^ satisfying ∑_*j*=1_^*l*(*i*)^ *c*_*j*_^(*i*)^ = 1. In addition, all state trajectories can be expressed as the state transition diagram with 2^3^ nodes.

In PBNs, we suppose that selecting one Boolean function is probabilistically independent at each time. However, it will be natural to consider the situation that switches of Boolean functions do not occur frequently. From this viewpoint, a context-sensitive PBN (CS-PBN) has been proposed in [13, 14]. In CS-PBNs, the deciding time of Boolean functions is also selected randomly. Hereafter, let *q* ∈ [0,1] denote the probability that Boolean functions are switched at time *k*, and a pair of the system (1) and *q* is called a CS-PBN.

**Figure 2:**
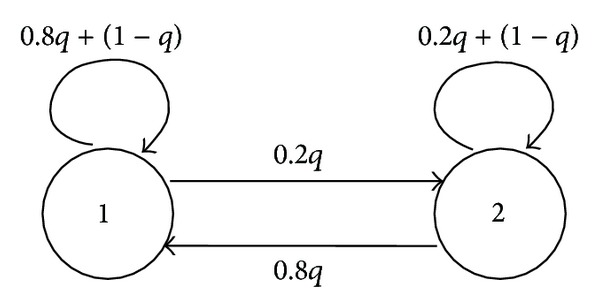
Discrete-time Markov chain in *f* ^(3)^.

To compare CS-PBNs with PBNs, consider (7) as a simple example. In PBNs, a switch of *f*_1_^(3)^ and *f*_2_^(3)^ functions does not depend on the Boolean function at time *k* − 1. In CS-PBNs, a switch of *f*_1_^(3)^ and *f*_2_^(3)^ is decided by the discrete-time Markov chain in Figure 2. In other words, this switch depends on the Boolean function at time *k* − 1. Owing to this difference, a control/verification method for CS-PBNs cannot be directly derived from that for PBNs.

### 2.2. Verification Using Model Checking

First, the reachability problem is formulated as the verification problem studied in this paper. The reachability problem is one of the typical verification problems. For a given CS-PBN, the output $y(k)={\left[\begin{array}{cccc} y_{1}(k) & y_{2}(k) & \cdots & y_{p}(k) \end{array}\right]}^{⊤}\in {\left\{0,1\right\}}^{p}$ is defined, where *y*_*i*_ = *x*_*j*_, *i* = 1,2,…, *p*, *j* ∈ *𝒥*⊆{1,2,…, *n*}. We remark that the output does not mean the measured signal. First, the reachability problem is formulated as follows.

**Problem 2 (reachability problem)**

Suppose that, for CS-PBN with the output, the initial state *x*(0) = *x*_0_, the initial Boolean function *f* ^(*i*)^(0, *x*(0), *u*(0)) = *f*_*j*_0_(*i*)_^(*i*)^(*x*(0), *u*(0)) (*j*_0_(*i*)∈{1,2,…, *l*(*i*)}), the control time *N*, and the target output *y*_*f*_ are given (*u*(0) is not given). Then, find a maximum probability *P*_max⁡_ that *y*(*k*) = *y*_*f*_ holds within time *N* by manipulating a control input sequence *u*(0), *u*(1),…, *u*(*N* − 1).

In the standard reachability problem, only terminal time is focused, and it is checked whether *y*(*N*) = *y*_*f*_ holds or not. In this paper, we focus on not only terminal time *N* but also other times 0,1,…, *N* − 1. Furthermore, since a CS-PBN has the control input, which can be regarded as a nondeterministic variable, we find a maximum probability satisfying the condition.

Next, we will propose a solution method for Problem 2. As a preparation, the following lemma [28] is introduced.

**Lemma 3**

Consider two binary variables *δ*_1_, *δ*_2_. Then, the following relations hold.

- ¬*δ*_1_ is equivalent to 1 − *δ*_1_.
- *δ*_1_∨*δ*_2_ is equivalent to *δ*_1_ + *δ*_2_ − *δ*_1_ *δ*_2_.
- *δ*_1_∧*δ*_2_ is equivalent to *δ*_1_ *δ*_2_.

For example, *δ*_1_∨¬*δ*_2_ is equivalently transformed into *δ*_1_ + (1 − *δ*_2_) − *δ*_1_(1 − *δ*_2_) = 1 − *δ*_2_ + *δ*_1_ *δ*_2_. By using this lemma, a Boolean function can be transformed into a polynomial on the real number field.

To solve Problem 2, the probabilistic model checker PRISM [15] is used. PRISM supports a discrete-time Markov chain (DT-MC), a continuous-time Markov chain (CT-MC), and a Markov decision process (MDP). PRISM consists of three parts: “Model,” “Properties,” and “Simulator.” In the “Model” part, a given probabilistic system is described using the PRISM language. In the “Properties” part, the property specification language incorporates temporal logic such as PCTL (probabilistic computation tree logic) [29], and we can verify if a given PCTL formula holds. In the “Simulator,” the state trajectories can be simulated.

**Figure 3:**
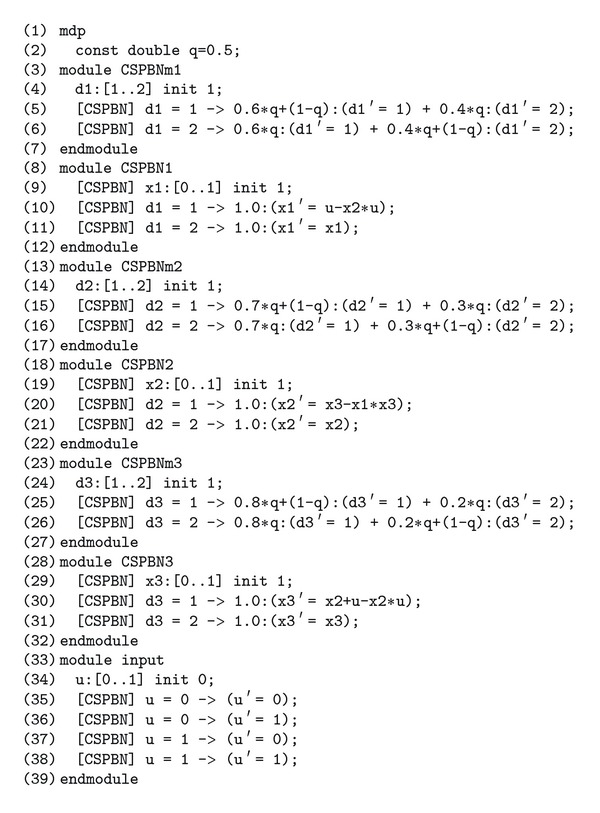
PRISM code expressing the CS-PBN.

Now, using PRISM, we propose a method for modeling a given CS-PBN. By modeling a given CS-PBN via PRISM, Problem 2 can be solved. In the PRISM-based method, Boolean functions in a given PBN can be directly used. To explain the PRISM-based method, consider the PBN (5)–(7) in Example 1 and *q* = 0.5. Suppose that the initial state and the initial Boolean function are given by $x_{0}={\left[\begin{array}{ccc} 1 & 1 & 1 \end{array}\right]}^{⊤}$, *f* ^(1)^(0) = *f*_1_^(1)^, *f* ^(2)^(0) = *f*_1_^(2)^, and *f* ^(3)^(0) = *f*_1_^(3)^ (i.e., *j*_0_(1) = *j*_0_(2) = *j*_0_(3) = 1), respectively. By using Lemma 3, each Boolean function can be transformed into some polynomial on the field of real numbers. Then, the PRISM code describing this CS-PBN is shown in Figure 3.

In line 1, it is described that a given system is a MDP; that is, the control input (in other words, the nondeterministic variable) that must decide is included. In line 2, the probability *q* is given by *q* = 0.5. In lines 3–7, the discrete-time Markov chain such as Figure 2 is modeled for *f* ^(1)^. The probabilistic variable d1 corresponds to *j* ∈ {1,2} in *f*_*j*_^(*i*)^. In line 4, *f* ^(1)^(0) is given by *f* ^(1)^(0) = *f*_1_^(1)^. In lines 5-6, the behavior of d1 is modeled. In line 5, it is described that if *d*_1_ = 1 holds, then the next state *d*_1_′ is 1 with the probability 0.6*q* + (1 − *q*) and 2 with the probability 0.4*q*. In lines 8–12, *f* ^(1)^ is modeled. In line 9, it is described that *x*_1_ takes a binary value, and the initial value of *x*_1_ is given by 1. In line 10, *f*_1_^(1)^ is modeled. In line 11, *f*_2_^(1)^ is modeled. In a similar way, *f* ^(2)^ is modeled in lines 13–22, and *f* ^(3)^ is modeled in lines 23–32. In CS-PBNs, a discrete probabilistic distribution is given for each *f* ^(*i*)^. Hence, *f* ^(*i*)^, *i* = 1,2, 3, must be modeled separately. To associate with each module, [CSPBN] is described. Finally, in lines 33–39, the property of the control input is described as a nondeterministic variable. Note that the initial value of the control input *u*(0) must be given (see line 34). Hence, PRISM must be executed for two cases of *u*(0) = 0 and *u*(0) = 1.

The above explanation is the outline of the PRISM-based modeling method. Based on the above example, we propose a procedure for deriving the PRISM code expressing a general CS-PBN.

#### 2.2.1. Procedure for Modeling CS-PBNs

**Step 1**

Transform each Boolean function into a polynomial on the real number field by using Lemma 3. The obtained Boolean functions are denoted by ${\hat{f}}_{j}^{\left(i\right)}$.

**Step 2**

Describe that a given system is a MDP, and give *q*.

**Step 3**

Describe modules CSPBNm *i* and CSPBN *i*, *i* = 1,2,…, *n*, as follows:

 module CSPBNm *i*
   
*d*_*i*_  : [1..*l*(*i*)] init *j*_0_(*i*);
   [CSPBN] *d*_*i*_ = 1→*c*_1_^(*i*)^ *q* + (1 − *q*):(*d*_*i*_′ = 1) + *c*_2_^(*i*)^ *q* : (*d*_*i*_′ = 2)+⋯+*c*_*l*(*i*)_^(*i*)^ *q* : (*d*_*i*_′ = *l*(*i*));
   [CSPBN] *d*_*i*_ = 2 → *c*_1_^(*i*)^ *q* : (*d*_*i*_′ = 1) + *c*_2_^(*i*)^ *q* + (1 − *q*):(*d*_*i*_′ = 2)+⋯+*c*_*l*(*i*)_^(*i*)^ *q* : (*d*_*i*_′ = *l*(*i*));
   ⋮
   [CSPBN] *d*_*i*_ = *l*(*i*) → *c*_1_^(*i*)^ *q* : (*d*_*i*_′ = 1) + *c*_2_^(*i*)^ *q* : (*d*_*i*_′ = 2)+⋯+*c*_*l*(*i*)_^(*i*)^ *q* + (1 − *q*):(*d*_*i*_′ = *l*(*i*));
 endmodule
 module CSPBN *i*
   
*x*_*i*_:[0..1] init *x*_*i*_(0);
   [CSPBN] *d*_*i*_ = 1→1.0: ($x_{i}^{\prime }={\hat{f}}_{1}^{\left(i\right)}(x,u)$);
   [CSPBN] *d*_*i*_ = 2→1.0: ($x_{i}^{\prime }={\hat{f}}_{2}^{\left(i\right)}(x,u)$);
   ⋮
   [CSPBN] *d*_*i*_ = *l*(*i*)→1.0: ($x_{i}^{\prime }={\hat{f}}_{l(i)}^{\left(i\right)}(x,u)$);
 endmodule

**Step 4**

Describe the control input *u*_*i*_, *i* = 1,2,…, *m*, as follows:

 module input *i*
   
*u*_*i*_:[0..1] init *u*_*i*_(0);
   [PBN1] *u*_*i*_ = 0→(*u*_*i*_′ = 0)
   [PBN1] *u*_*i*_ = 0→(*u*_*i*_′ = 1)
   [PBN1] *u*_*i*_ = 1→(*u*_*i*_′ = 0)
   [PBN1] *u*_*i*_ = 1→(*u*_*i*_′ = 1)
 endmodule

Finally, consider solving Problem 2. For solving this problem, we use Pmax prepared in PRISM. For example, suppose that $y={\left[\begin{array}{cc} y_{1} & y_{2} \end{array}\right]}^{⊤}$ and $y_{f}={\left[\begin{array}{cc} 0 & 1 \end{array}\right]}^{⊤}$. Then, in PRISM, Problem 2 can be described by

(8) $$\begin{array}{c} Pmax=?\left[F<=N\;\;\left(y1=0\right)\&\left(y2=1\right)\right]. \end{array}$$

Therefore, we see that Problem 2 can be solved using PRISM. The control input sequence *u*(0), *u*(1),…, *u*(*N* − 1) is obtained simultaneously, but in PRISM 4.0.3, the obtained control input sequence cannot be displayed except for the case of *N* = *∞*. In the case of *N* = *∞*, the discrete-time Markov chain can be obtained as the closed-loop system of a given CS-PBN.

### 2.3. Optimal Control Using Polynomial Optimization

Consider the following problem.

**Problem 4 (optimal control problem)**

Suppose that, for CS-PBN, the initial state *x*(0) = *x*_0_, the initial Boolean function *f* ^(*i*)^(0, *x*(0), *u*(0)) = *f*_*j*_0_(*i*)_^(*i*)^(*x*(0), *u*(0))(*j*_0_(*i*)∈{1,2,…, *l*(*i*)}), and the control time *N* are given (*u*(0) is not given). Then, find a control input sequence *u*(0), *u*(1),…, *u*(*N* − 1) minimizing the cost function

(9) $$\begin{array}{c} J=E\left[\sum_{k=0}^{N-1}\left\{Qx\left(k\right)+Ru\left(k\right)\right\}+Q_{f}x\left(N\right)\;|\;x\left(0\right)=x_{0}\right], \end{array}$$

where *Q*, *Q*_*f*_ ∈ *ℛ* ^1×*n*^, *R* ∈ *ℛ* ^1×*m*^ are weighting vectors whose element is a nonnegative real number and *E*[·∣·] denotes a conditional expected value.

According to the following two reasons, the linear cost function (9) is appropriate. (i) For a binary variable *δ* ∈ {0,1}, the relation *δ* ^2^ = *δ* holds. That is, in the cost function, the quadratic term such as *x*_*i*_^2^(*k*) is not necessary. (ii) In control of GRNs, expression of a certain gene is frequently focused (see, e.g., [19]). That is, in the cost function, the quadratic term such as *x*_*i*_(*k*)*x*_*j*_(*k*), *i*≠*j*, is not necessary.

For a PBN, the authors have derived the following recursive representation of the expected value of the state:

(10) $$\begin{array}{c} E\left[x_{i}\left(k+1\right)\right]=\sum_{j=1}^{l(i)}c_{j}^{\left(i\right)}{\hat{f}}_{j}^{\left(i\right)}\left(E\left[x\left(k\right)\right],u\left(k\right)\right), \end{array}$$

where the condition *x*(0) = *x*_0_ in the expected value is omitted. See [23] for further details. In this paper, this representation is extended to that in CS-PBNs.

First, we present a simple example. Consider the PBN (5)–(7) in Example 1. Suppose that the initial state and the initial Boolean function are given by $x_{0}={\left[\begin{array}{ccc} 1 & 1 & 1 \end{array}\right]}^{⊤}$, *f* ^(1)^(0) = *f*_1_^(1)^, *f* ^(2)^(0) = *f*_1_^(2)^, and *f* ^(3)^(0) = *f*_1_^(3)^, respectively.

Consider deriving the expected value of the state at time *k* = 1. Suppose that *u*(0) = 0. Since the Boolean function at time *k* = 0 is given, the state at time *k* = 1 is uniquely derived as

(11) $$\begin{array}{c} E\left[x\left(1\right)\;|\;x\left(0\right)=x_{0},u\left(0\right)=0\right]=x\left(1\right)={\left[\begin{array}{ccc} 0 & 0 & 1 \end{array}\right]}^{⊤}. \end{array}$$

Hereafter, the condition such as *x*(0) = *x*_0_, *u*(0) = 0 in the expected value is omitted. Next, consider deriving the expected value of the state at time *k* = 2. We remark that, for each *x*_*i*_, the discrete-time Markov chain such as Figure 2 can be obtained. For example, the probability that *f*_1_^(1)^ is selected at time *k* = 1 is 0.6*q* + (1 − *q*), and the probability that *f*_2_^(1)^ is selected at time *k* = 1 is 0.4*q*. Suppose that *u*(1) = 1. Then, we can obtain

(12) E[x1(2)]=E[f(1)(1,x(1),u(1))]=(0.6q+(1−q))E[f1(1)(x(1),u(1))] +0.4qE[f2(1)(x(1),u(1))]=(0.6q+(1−q))·(1−0)·1+0.4q·0=0.6q+(1−q),E[x2(2)]=E[f(2)(1,x(1),u(1))]=(0.7q+(1−q))E[f1(2)(x(1),u(1))] +0.3qE[f2(2)(x(1),u(1))]=(0.7q+(1−q))·(1−0)·1+0.3q·0=0.7q+(1−q),E[x3(2)]=E[f(3)(1,x(1),u(1))]=(0.8q+(1−q))E[f1(3)(x(1),u(1))] +0.2qE[f2(3)(x(1),u(1))]=(0.8q+(1−q))·(0+1−0·1)+0.2q·1=1.0.

Finally, we remark that the probability that some Boolean function *f*_*j*_^(*i*)^ is selected is time-varying. For example, the probability that *f*_1_^(1)^ is selected at time *k* = 2 is (0.6*q* + (1 − *q*))^2^ + 0.4*q* · 0.6*q*, and the probability that *f*_2_^(1)^ is selected at time *k* = 2 is (0.6*q* + (1 − *q*)) · 0.4*q* + 0.4*q* · (0.2*q* + (1 − *q*)).

Next, consider a general case. From the observation of the above example, we can obtain the following recursive representation:

(13) $$\begin{array}{c} E\left[x_{i}\left(k+1\right)\right]=\sum_{j=1}^{l(i)}d_{j}^{\left(i\right)}\left(k\right){\hat{f}}_{j}^{\left(i\right)}\left(E\left[x\left(k\right)\right],u\left(k\right)\right),\\ d^{\left(i\right)}\left(k+1\right)=P^{\left(i\right)}d^{\left(i\right)}\left(k\right), \end{array}$$

where $d^{\left(i\right)}(k)={\left[\begin{array}{cccc} d_{1}^{\left(i\right)}(k) & d_{2}^{\left(i\right)}(k) & \cdots & d_{l(i)}^{\left(i\right)}(k) \end{array}\right]}^{⊤}$ and

(14) $$\begin{array}{c} P^{\left(i\right)}=\left[\begin{array}{cccc} c_{1}^{\left(i\right)}q+\left(1-q\right) & c_{2}^{\left(i\right)}q & \cdots & c_{l(i)}^{\left(i\right)}q\\ c_{1}^{\left(i\right)}q & c_{2}^{\left(i\right)}q+\left(1-q\right) & \cdots & c_{l(i)}^{\left(i\right)}q\\ \vdots & \vdots & \ddots & \vdots \\ c_{1}^{\left(i\right)}q & c_{2}^{\left(i\right)}q & \cdots & c_{l(i)}^{\left(i\right)}q+\left(1-q\right) \end{array}\right]. \end{array}$$

Therefore, Problem 4 can be reduced to the following polynomial optimization problem:

(15) find    E[x(k+1)]∈ℛn,  u(k)∈ℛm,k=0,1,…,N−1,min⁡   Cost  function(9),subject  to System(13), i=1,2,…,n,      x(0)=x0,      ui(k)(ui(k)−1)=0.

The constraint *u*_*i*_(*k*)(*u*_*i*_(*k*) − 1) = 0 guarantees that *u*(*k*) is a binary variable. A polynomial optimization problem can be solved by using a suitable solver such as SparsePOP [30].

## 3. Results and Discussion

In this section, we present a numerical example on the WNT5A network. First, the WNT5A network is explained. Next, computational results are presented.

### 3.1. WNT5A Network

Consider the GRN with the gene WNT5A, which is related to melanoma. A Boolean network model is given by

(16) x1(k+1)=¬x6(k),x2(k+1)=(¬x2(k)∧x4(k)∧x6(k)) ∨{x2(k)∧(x4(k)∨x6(k))},x3(k+1)=¬x7(k),x4(k+1)=x4(k),x5(k+1)=x2(k)∨¬x7(k),x6(k+1)=x3(k)∨x4(k),x7(k+1)=¬x2(k)∨x7(k),

where the concentration level (high or low) of the gene WNT5A is denoted by *x*_1_, the concentration level of the gene pirin by *x*_2_, the concentration level of the gene S100P by *x*_3_, the concentration level of the gene RET1 by *x*_4_, the concentration level of the gene MART1 by *x*_5_, the concentration level of the gene HADHB by *x*_6_, and the concentration level of the gene STC2 by *x*_7_. See [31] for further details.

Next, suppose that the control input *u* is given by *x*_2_ (the concentration level of the gene pirin), according to the discussion in [19]. By replacing *x*_2_ and *x*_3_, *x*_4_,…, *x*_7_ with *u* and *x*_2_, *x*_3_,…, *x*_6_, respectively, we can obtain the following model:

(17) $$\begin{array}{c} x_{1}\left(k+1\right)=f_{d}^{\left(1\right)}\left(x\left(k\right),u\left(k\right)\right)=\neg x_{5}\left(k\right),\\ x_{2}\left(k+1\right)=f_{d}^{\left(2\right)}\left(x\left(k\right),u\left(k\right)\right)=\neg x_{6}\left(k\right),\\ x_{3}\left(k+1\right)=f_{d}^{\left(3\right)}\left(x\left(k\right),u\left(k\right)\right)=x_{3}\left(k\right),\\ x_{4}\left(k+1\right)=f_{d}^{\left(4\right)}\left(x\left(k\right),u\left(k\right)\right)=\neg x_{6}\left(k\right)\vee u\left(k\right),\\ x_{5}\left(k+1\right)=f_{d}^{\left(5\right)}\left(x\left(k\right),u\left(k\right)\right)=x_{2}\left(k\right)\vee x_{3}\left(k\right),\\ x_{6}\left(k+1\right)=f_{d}^{\left(6\right)}\left(x\left(k\right),u\left(k\right)\right)=x_{6}\left(k\right)\vee \neg u\left(k\right). \end{array}$$

Furthermore, we add the probabilistic behavior as follows:

(18) xi(k+1)  ={fd(i)(x(k),u(k)),with  the  probability  c1,xi(k),with  the  probability  c2,

where *l*(*i*) = 2 holds. Thus, we can obtain the PBN model expressing a WNT5A network.

### 3.2. Computational Result on Verification

Consider solving Problem 4. For the PBN (18), we assume that *c*_1_ and *c*_2_ are given by *c*_1_ = 0.5 and *c*_2_ = 0.5, respectively. In the WNT5A network, it is important to inhibit the concentration level *x*_1_ of the gene WNT5A [32]. From this fact, we set *y* = *x*_1_ and *y*_*f*_ = 0. The initial state is given by $x_{0}={\left[\begin{array}{cccccc} 1 & 0 & 0 & 1 & 0 & 0 \end{array}\right]}^{⊤}$. The initial Boolean function is given by *f*_*d*_^(*i*)^. In addition, we set *N* = 5.

Next, we show the computation result. Then, we can obtain *P*_max⁡_ = 0.7215 for *q* = 0.3, *P*_max⁡_ = 0.6587 for *q* = 0.5, and *P*_max⁡_ = 0.6489 for *q* = 0.7. It is desirable that *P*_max⁡_ is close to 1. Hence, we see that the performance is degraded for a larger *q*.

### 3.3. Computational Result on Optimal Control

Consider solving Problem 4. For the PBN (18), we assume that *c*_1_ and *c*_2_ are given by *c*_1_ = 0.8 and *c*_2_ = 0.2, respectively. Since the concentration level *x*_1_ must be inhibited, the weights *Q*, *Q*_*f*_, and *R* in Problem 4 are given by

(19) $$\begin{array}{c} Q=\left[\begin{array}{cccccc} 1 & 0 & 0 & 0 & 0 & 0 \end{array}\right],\;\;R=1,\\ Q_{f}=\left[\begin{array}{cccccc} 10 & 0 & 0 & 0 & 0 & 0 \end{array}\right], \end{array}$$

respectively. The initial state is given by $x_{0}={\left[\begin{array}{cccccc} 1 & 1 & 0 & 1 & 0 & 0 \end{array}\right]}^{⊤}$. The initial Boolean function is given by *f*_*d*_^(*i*)^. In addition, we set *N* = 5 and *q* = 0.3.

Next, we show the computation result. By solving Problem 4, we can obtain *u*(0) = *u*(1) = 1, *u*(2) = *u*(3) = *u*(4) = 0. The expected value of the state at each time is obtained as

(20) $$\begin{array}{c} E\left[x\left(1\right)\right]={\left[\begin{array}{cccccc} 1 & 1 & 0 & 1 & 1 & 0 \end{array}\right]}^{⊤},\\ E\left[x\left(2\right)\right]={\left[\begin{array}{cccccc} 0.06 & 1 & 0 & 1 & 1 & 0 \end{array}\right]}^{⊤},\\ E\left[x\left(3\right)\right]={\left[\begin{array}{cccccc} 0.0061 & 1 & 0 & 1 & 1 & 0 \end{array}\right]}^{⊤},\\ E\left[x\left(4\right)\right]={\left[\begin{array}{cccccc} 0.0008 & 0.22 & 0 & 0.22 & 1 & 0.9866 \end{array}\right]}^{⊤},\\ E\left[x\left(5\right)\right]={\left[\begin{array}{cccccc} 0.0001 & 0.0448 & 0 & 0.0448 & 0.3385 & 0.998 \end{array}\right]}^{⊤}. \end{array}$$

Hence, we see that the concentration level *x*_1_ of the gene WNT5A is inhibited with time.

In addition, the optimal value J* of the cost function was 5.23. For *q* = 0.5 and *q* = 0.7, we can obtain J* = 5.41 and J* = 5.57, respectively. From these values, we see that the performance is degraded for a larger *q*.

## 4. Conclusions

In this paper, we discussed verification and optimal control for a context-sensitive probabilistic Boolean network (CS-PBN), which is one of the models for gene regulatory networks (GRNs). In verification, the PRISM-based method for PBNs [16] was extended to that for CS-PBNs. In optimal control, the optimal control method for PBNs [23] was extended to that for CS-PBNs. A CS-PBN is a generalized version of a PBN, and it enables us to consider several situations. Furthermore, as a numerical example, we considered the WNT5A network, which is related to melanoma. The proposed methods provide us useful tools in control theory of GRNs.

In recent years, a stochastic Boolean network [33] has been proposed as a new representation of PBNs. In addition, to simplify a given Boolean network, the Karnaugh map realization of a Boolean network has been proposed in [34]. These modeling methods will be useful for reducing the computational burden. Future efforts will focus on applying these modeling methods to the control problem and the verification for CS-PBNs.
